# Lipids profile among ART-naïve HIV infected patients and men who have sex with men in China: a case control study

**DOI:** 10.1186/s12944-016-0297-1

**Published:** 2016-09-06

**Authors:** Qi Wang, Haibo Ding, Junjie Xu, Wenqing Geng, Jing Liu, Xiaolin Guo, Jing Kang, Xiaolin Li, Yongjun Jiang, Hong Shang

**Affiliations:** 1 Key Laboratory of AIDS Immunology of National Health and Family Planning Commission, Department of Laboratory Medicine, The First Affiliated Hospital, China Medical University, Shenyang, Liaoning 110001 People’s Republic of China; 2 Collaborative Innovation Center for Diagnosis and Treatment of Infectious Diseases, Hangzhou, China

**Keywords:** Human immunodeficiency virus (HIV), Atherogenic index of plasma (AIP), Lipids, Dyslipidemia

## Abstract

**Background:**

Dyslipidemia is commonly seen in human immunodeficiency virus (HIV) infected patients. Understanding the risk factors of abnormal lipid profiles is urgent for proposing targeted approaches to prevention. Our objective was to assess the incidence and associated factors of abnormal lipid profiles and atherogenic index of plasma (AIP) among antiretroviral therapy (ART) naïve men who have sex with men (MSM) acute HIV infection (AHI) and chronic HIV infection (CHI) patients in China.

**Methods:**

We compared lipids parameters such as triglycerides (TG), low-density lipoprotein cholesterol (LDL-C), total cholesterol (TC), high-density lipoprotein cholesterol (HDL-C) and AIP between MSM HIV-infected patients and MSM HIV negative controls. Multivariable linear regression was used to evaluate risk factors of higher AIP.

**Results:**

We performed a case control analysis of 110 AHI, 110 CHI and 100 HIV negative MSM participants. The TC, HDL-C and LDL-C levels were decreased in the AHI and CHI groups compared to the controls (3.90 ± 0.73 mmol/L and 3.72 ± 0.74 mmol/L versus 4.49 ± 0.91 mmol/L, *p* < 0.001; 1.00 ± 0.25 mmol/L and 1.01 ± 0.30 mmol/L versus 1.19 ± 0.29 mmol/L, *p* < 0.001; 2.11 ± 0.57 mmol/L and 2.22 ± 0.58 mmol/L versus 2.75 ± 0.78 mmol/L, *p* < 0.001). The AIP score was higher in the AHI patients compared to the control group [0.08 (−0.05–0.20) versus−0.04 (−0.21–0.22), *p* = 0.039]. In total groups, AIP was associated with AHI and TG positively (β = 0.029 ± 0.012, *p* = 0.015;β = 0.273 ± 0.009, *p* < 0.001) and correlated with HDL-C inversely (β = -0.444 ± 0.023, *p* < 0.001).

**Conclusions:**

HIV infection contributed to decreased TC, LDL-C and HDL-C. AHI contributed to higher AIP level. An urgent need exists for earlier HIV diagnosis and better prevention of dyslipidemia in China.

## Background

As a kind of metabolic abnormalities, dyslipidemia has been of significant concern among human immunodeficiency virus (HIV) infected patients [1–3]. HIV-infected adults with lipids perturbations may be at risk of accelerated atherosclerosis and cardiovascular disease [4]. Various lipid abnormalities have been recorded in HIV patients with antiretroviral therapy (ART), particularly those on protease inhibitors based regimes [5, 6]. The dyslipidemia associated with ART is manifested as increased trigylcerides (TG), low-density lipoprotein cholesterol (LDL-C), total cholesterol (TC) and lower high-density lipoprotein cholesterol (HDL-C) typically [7, 8].

Recently, the effect of HIV itself has been proposed as a contributor to lipid abnormalities. HIV infection may lead to structural and functional changes in HDL-C [9]. In both ART‑naïve chronic HIV infection (CHI) adult and children patients, low HDL-C was the most frequently observed abnormality and there existed a significant relationship between lipid parameters and viral load [10, 11]. Results from the Veterans Aging Cohort Study showed that HIV infection was associated with decreased TC, HDL-C, LDL-C and elevation of TG, ART initiation did not reverse alteration in TG or LDL-C to pre-HIV infection levels [12]. This suggests that HIV infection may influence lipids regardless of ART status. Furthermore, experiment in vitro demonstrated that the high viremia levels of acute HIV infection (AHI) impaired cholesterol efflux capacity, which indicated that dyslipidemia may occur very early [13].

However, relying solely on single lipid parameter is not sufficient for evaluating lipid metabolism. Attentions should be paid to more sensitive indexes. The atherogenic index of plasma (AIP) [log(TG/HDL-C)], which correlates well with the size of HDL-C and LDL-C particles, reflects the presence of atherogenic small particles accurately [14]. AIP is scarcely taken into consideration in studies which investigating lipid profiles in HIV patients. In recent years, men who have sex with men (MSM) have been a major core risk population for HIV epidemic in China [15]. Moreover, some traditional risk factors for dyslipidemia such as smoking, alcohol, abnormal nutritional status, and recreational drug use were more likely to be prevalent among MSM [16, 17]. Thus, we hypothesize that the proportion of abnormal lipid profiles in ART‑naïve MSM HIV patients in China is high and may occur very early.

Therefore, we investigated the condition of lipid metabolism and AIP among AHI and CHI ART-naïve MSM populations in China without the confounding factor of ART with a well matched HIV negative MSM group. Furthermore, we aimed to investigate the contribution of HIV to lipid abnormalities and associated factors influencing AIP in ART-naïve patients in China.

## Methods

### Study design and participants

This was a cross‑sectional study carried out at the acquired immune deficiency syndrome (AIDS) clinical care centre of The First Affiliated Hospital of China Medical University and the Beijing You’An Hospital from January 2013 to June 2015 consecutively. Every 4–8 weeks, 6 000 individuals from the HIV primary open cohort in Beijing and 1650 volunteers recruited from the Rainbow Harbor voluntary counseling and testing (VCT) Center in the First Affiliated Hospital of China Medical University in Shenyang were tested for AHI. The plasmas were collected and tested for HIV antibodies and HIV viral load. Participants were classified as AHI who met 1 of 2 criteria: (1) had negative or uncertain HIV serology with HIV RNA more than 10,000 copies/ml or (2) had a positive HIV serology with evidence of negative testing in the previous 6 months. Other volunteers who were HIV negative were invited in the control group (no-HIV group). The inclusion criteria for the no-HIV group were (a) male, self-reporting anal intercourse with male partners in the past 1 year, (b) aged 18 years or older. Eligibility criterion for the CHI group was positive HIV antibody confirmed by Western blot test for more than 6 months.

Exclusion criteria for the three groups were suffering with severe respiratory failure, kidney failure, heart failure (New York class III or IV), chronic alcoholism, cachexia status, diabetes, taking medicines of steroid hormones or lipid-lowing drugs at the study recruitment period. The study was approved by the Ethics Committee of the First Affiliated Hospital of China Medical University and the Beijing You’An Hospital. All participants provided written informed consent before attending this study.

### Data collection

Data regarding sociodemographic characteristics, including gender, age, height, weight, smoking, alcohol consumption were recorded through a interviewer filled questionnaire which administered face to face in a private counseling room. The body mass index (BMI) was calculated using the formula of weight (kg)/height (m^2^)﻿*10000.

Venous blood was drawn by trained nurses. Serum samples were tested for HIV antibody screening using a third-generation enzyme-linked immunosorbent assay. Samples with positive screening test result were confirmed by HIV-1/2 Western blot assay. We used the whole blood samples to determine the counts and percentages of CD4 + T cells and CD8 + T cells by a FACS Calibur flow cytometer (Becton-Dickinson, USA). Plasma HIV viral load was detected by Roche COBAS TaqMan.

The blood pressure was measured by a sphygmomanometer with the patient sitting quietly. The systolic blood pressure (SBP) is equivalent to the value of mercury column when hearing the first sound of artery pulse. When the artery pulse sound decreased or disappeared, the reading of the mercury column is the value of diastolic blood pressure (DBP). The blood pressure was measured twice at least 5 min apart. The mean value of two results was recorded. Hypertension was defined as SBP≧140 mmHg and/or DBP≧90 mmHg [18].

Fasting blood glucose (FBG) and lipids were measured by Roche D/P/P modular analysis system using enzymatic method. Any kind of the abnormal lipid profiles was defined as dyslipidemia according to the World Health Organization (WHO) criteria thus hypertriglyceridemia (TG > 1.7 mmol/L), hypercholesterolemia (TC > 5.2 mmol/L), low HDL-C (HDL-C < 0.9 mmol/L), high LDL-C (LDL-C > 3.5 mmol/L), and high TC/HDL-C ratio (>5) [19]. The ratios of TC/HDL-C and AIP were calculated. The ratio log (TG/HDL-C), which is called AIP, was defined abnormal when >0.1 [20].

### Statistical analysis

All analyses were performed by using SPSS version 18.0 software. Continuous variables were described as mean and standard deviation (SD) or median and interquartile range (IQR). Chi‑square test was used to test for the difference between categorical variables, whereas the difference between continuous variables was analyzed using the one way ANOVA as appropriate. Pearson’s correlation coefficient was used to assess the correlations between AIP and clinical parameters. Univariable linear regression (enter) was performed to identify variables predictive of AIP. All variables with *p*-values < 0.1 were included in a multivariable linear regression model to examine the potential determinants. A two-tailed *p*-value less than 0.05 was considered statistically significant.

## Results

During the study period, 100 cases of the no-HIV control group, 110 AHI and 110 CHI patients were enrolled. Demographic and clinical characteristics of the study population were summarized in Table 1. The mean age of AHI group, CHI group and no-HIV control group were 31.5 ± 8.6, 33.8 ± 6.2 and 32.2 ± 9.0 respectively. There were no statistically significant differences between the three groups in terms of age, height, weight, BMI, SBP, DBP and proportion of smoking, drinking, hypertension. The mean FBG of the CHI group was statistically higher than that of the no-HIV control group and the AHI group (*p* < 0.05). Compared with the CHI group, the AHI group had shorter estimated infection time [65.0 (38.5–106.0) days vs. 631.0 (133.0–1135.0) days, *p* < 0.001] and higher CD4 cell counts [404.0 (281.5–531.5) cell/μl vs. 173.0 (113.3–243.2) cell/μl, *p* < 0.001]. The viral load level between AHI and CHI groups had no statistically difference.

**Table 1:** Demographic and clinical characteristics of the no-HIV and AHI and CHI participants

Variables	No-HIV (*n* = 100)	AHI (*n* = 110)	CHI (*n* = 110)
Age (years)	32.2 ± 9.0	31.5 ± 8.6	33.8 ± 6.2
Height (cm)	174.0 ± 5.1	173.9 ± 4.6	174.5 ± 6.0
Weight (kg)	65.9 ± 11.4	63.9 ± 8.5	66.1 ± 10.9
BMI (kg/m^2^)	21.8 ± 3.6	21.1 ± 2.6	21.7 ± 3.4
History of smoking, *n* (%)	38 (38.0)	29 (26.4)	37 (33.6)
History of alcohol, *n* (%)	45 (45.0)	44 (40.0)	52 (47.3)
SBP (mmHg)	120.0 (114.6–122.7)	114.5 (112.0–120.0)	120.0 (116.0–126.1)
DBP (mmHg)	80.0 (79.0–84.2)	76.0 (72.0–78.2)	83.6 (79.1–88.0)
Hypertension, *n* (%)	17 (17.0)	10 (9.1)	19 (17.3)
FBG (mmol/L)	4.59 (4.21–5.01)	4.88 (4.47–5.27)	5.13 (4.76–5.53)^a^
Estimated infection (days)	NA	65.0 (38.5–106.0)^b^	631.0 (133.0–1135.0)
CD4 cell count (cells/mm^3^)	NA	404.0 (281.5–531.5)^b^	173.0 (113.3–243.2)
HIV RNA (log10 copies/ml)	NA	4.83 (4.43–5.27)	4.66 (4.27–4.88)

*No*-*HIV* HIV negative control, *AHI* acute HIV infection group, *CHI* chronic HIV infection group, *n* indicates the number of participants, *BMI* body mass index, *SBP* brachial systolic blood pressure, *DBP* brachial diastolic blood pressure, *FBG* fasting blood glucose

Data are expressed as mean with ± standard error of mean, geometric mean with 5^th^ and 95^th^ percentile intervals, *n* or % of *n*

*NA* None applicable

^a^ *p* value < 0.05 between CHI and AHI, CHI and no-HIV

^b^ *p* value < 0.05 between AHI and CHI

**Fig. 1:**
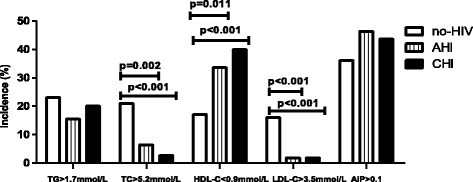
Incidence of undesirable lipid concentrations among the no-HIV, AHI and CHI group. No-HIV, HIV negative control ; AHI, acute HIV infection group; CHI, chronic HIV infection group; TG, triglycerides; TC, total cholesterol; HDL-C, high-density lipoprotein cholesterol; LDL-C, low-density lipoprotein cholesterol; AIP, the atherogenic index of plasma

The median value and abnormal proportion of TC, LDL-C of the no-HIV control group were higher than the other two groups (Table 2 and Fig. 1). The no-HIV control group had the highest level of HDL-C and the abnormal proportion of HDL-C of the no-HIV control group was lower than the other two groups. We found similar values of TG, TC/HDL-C, proportion of hypertriglyceridemia, high TC/HDL-C, and dyslipidemia for the three groups. Compared with the no-HIV control group, the AHI group had higher AIP score [0.08 (−0.05–0.20) vs.−0.04 (−0.21–0.22), *p* = 0.039]. There was no difference in the proportion of abnormal AIP of the three groups.

**Table 2:** Lipid profiles and AIP of the no-HIV, AHI and CHI participants

Variables	No-HIV (*n* = 100)	AHI (*n* = 110)	CHI (*n* = 110)
TG (mmol/L)	1.17 (0.76–1.67)	1.11 (0.89–1.52)	1.06 (0.79–1.42)
TC (mmol/L)	4.49 ± 0.91^a^	3.90 ± 0.73	3.72 ± 0.74
HDL-C (mmol/L)	1.19 ± 0.29^a^	1.00 ± 0.25	1.01 ± 0.30
LDL-C (mmol/L)	2.75 ± 0.78^a^	2.11 ± 0.57	2.22 ± 0.58
TC/HDL-C	3.79 (3.18–4.54)	3.95 (3.45–4.57)	3.67 (3.19–4.29)
Hypertriglyceridemia, *n* (%)	23 (23.0)	17 (15.5)	22 (20.0)
Hypercholesterolemia, *n* (%)	21 (21.0)^a^	7 (6.4)	3 (2.7)
Low HDL-C, *n* (%)	17 (17.0)^a^	37 (33.6)	44 (40.0)
High LDL-C, *n* (%)	16 (16.0)^a^	2 (1.8)	2 (1.8)
High TC/HDL-C ratio, *n* (%)	16 (16.0)	16 (14.5)	15 (13.6)
Dyslipidemia, *n* (%)	44 (44.0)	51 (46.4)	52 (47.3)
AIP	−0.04 (−0.21–0.22)	0.08 (−0.05–0.20)^b^	0.03 (−0.17–0.23)
AIP > 0.1, *n* (%)	38 (38.0)	51 (46.4)	48 (43.6)

*No*-*HIV* HIV negative control, *AHI* acute HIV infection group, *CHI* chronic HIV infection group, *TG* triglycerides, *TC* total cholesterol, *HDL*-*C* high-density lipoprotein cholesterol, *LDL*-*C* low-density lipoprotein cholesterol, *AIP* the atherogenic index of plasma

^a^ *p* value < 0.05 between no-HIV and AHI, no-HIV and CHI

^b^ *p* value < 0.05 between no-HIV and AHI

Using Pearson’s correlation coefficient, AIP was positively correlated with TC (*p* = 0.009), LDL-C (*p* = 0.014), weight (*p* = 0.027), BMI (*p* = 0.018), DBP (*p* = 0.045) in the no-HIV group (Table 3, Model 1). HIV specific sub-analysis was performed in 220 cases of HIV infected patients (AHI group and CHI group), we found that weight (*p* = 0.001), BMI (*p* < 0.001) and HIV RNA (*p* = 0.029) were positively correlated with AIP (Table 3, Model 2).

**Table 3:** Pearson correlation coefficients between AIP and clinical and laboratory factors in the no-HIV group (Model 1), and in the HIV group (Model 2)

	Model 1		Model 2*	
Factors	R	*P*	R	*P*
TG (mmol/l)	0.915	<0.001	0.843	<0.001
TC (mmol/l)	0.258	0.009	−0.015	0.821
HDL-C (mmol/l)	−0.680	<0.001	−0.653	<0.001
LDL-C (mmol/l)	0.245	0.014	0.021	0.751
FBG (mmol/l)	0.086	0.397	0.049	0.473
Age (years)	0.172	0.087	0.124	0.066
Height (cm)	0.002	0.987	0.001	0.990
Weight (kg)	0.221	0.027	0.219	0.001
BMI (kg/m^2^)	0.236	0.018	0.233	<0.001
SBP (mmHg)	0.155	0.123	0.095	0.162
DBP (mmHg)	0.201	0.045	0.118	0.082
CD4 cell count (cells/mm^3^)	--	--	−0.063	0.364
HIV RNA (log10 copies/ml)	--	--	0.151	0.029

Model 2*, AHI and CHI patients were all included

*TG* triglycerides, *TC* total cholesterol, *HDL*-*C* high-density lipoprotein cholesterol, *LDL*-*C* low-density lipoprotein cholesterol, *FBG* fasting blood glucose, *BMI* body mass index, *SBP* brachial systolic blood pressure, *DBP* brachial diastolic blood pressure

Multiple linear regression analysis was carried out to determine the significant predictors of AIP in different groups (Table 4). In no-HIV control group, AIP was associated with TG and TC positively (β = 0.245 ± 0.013, *p* < 0.001;β = 0.039 ± 0.013, *p* = 0.003) and correlated with HDL-C inversely (β =−0.413 ± 0.041, *p* < 0.001). In the AHI group, significant correlation was noticed between AIP and TG (β = 0.353 ± 0.010, *p* < 0.001), HDL-C (β =−0.417 ± 0.018, *p* < 0.001). In the CHI group we found that TG (β = 0.214 ± 0.018, *p* < 0.001), TC (β = 0.138 ± 0.049, *p* = 0.006) and LDL-C (β = 0.100 ± 0.048, *p* = 0.040) were positively correlated with AIP and significant inversely correlation was noticed between AIP and HDL-C (β =−0.638 ± 0.066, *p* < 0.001). In total groups, AIP was associated with AHI and TG positively (β = 0.029 ± 0.012, *p* = 0.015;β = 0.273 ± 0.009, *p* < 0.001 ) and correlated with HDL-C inversely (β =−0.444 ± 0.023, *p* < 0.001 ).

**Table 4:** Factors correlated with AIP after controlling for potential confounders

	No-HIV		AHI		CHI		Total*	
	β ± SE	*p*	β ± SE	*p*	β ± SE	*p*	β ± SE	*p*
AHI versus HIV-negative	---	---	---	---	---	---	0.029 ± 0.012	0.015
CHI versus HIV-negative	---	---	---	---	---	---	---	---
Age (years)	0.001 ± 0.001	0.322	−0.001 ± 0.001	0.209	−0.001 ± 0.002	0.747	−0.001 ± .001	0.343
Smoker versus nonsmoker	−0.004 ± 0.022	0.841	0.009 ± 0.010	0.374	0.020 ± 0.022	0.369	0.013 ± 0.013	0.319
Drinker versus nondrinker	0.009 ± 0.020	0.653	−0.012 ± 0.009	0.177	−0.033 ± 0.022	0.137	−0.011 ± 0.012	0.342
BMI (kg/m^2^)	−0.001 ± 0.003	0.690	0.001 ± 0.002	0.520	0.000 ± 0.003	0.963	0.003 ± 0.002	0.139
TG (mmol/L)	0.245 ± 0.013	<0.001	0.353 ± 0.010	<0.001	0.214 ± 0.018	<0.001	0.273 ± 0.009	<0.001
TC (mmol/L)	0.039 ± 0.013	0.003	---	---	0.138 ± 0.049	0.006	---	---
HDL-C (mmol/L)	−0.413 ± 0.041	<0.001	−0.417 ± 0.018	<0.001	−0.638 ± 0.066	<0.001	−0.444 ± 0.023	<0.001
LDL-C (mmol/L)	---	---	---	---	0.100 ± 0.048	0.040	---	---
FBG (mmol/L)	---	---	---	---	---	---	---	---
SBP (mmHg)	---	---	---	---	---	---	---	---
DBP (mmHg)	---	---	---	---	---	---	---	---
CD4 cell count (cells/mm3)	---	---	---	---	---	---	---	---
HIV RNA (log10 copies/ml)	---	---	---	---	---	---	---	---

*No*-*HIV* HIV negative control, *AHI* acute HIV infection group, *CHI* chronic HIV infection group, Total*, no-HIV, AHI and CHI participants were all included; *BMI* body mass index, *TG* triglycerides, *TC* total cholesterol, *HDL*-c high-density lipoprotein cholesterol, *LDL*-*C* low-density lipoprotein cholesterol, *FBG* fasting blood glucose, *SBP* brachial systolic blood pressure, *DBP* brachial diastolic blood pressure

The β values are the multivariate regression unstandardized coefficients

*SE*, standard error of mean

## Discussion

Our observational study comprehensively compared the lipid levels and AIP among ART-naïve MSM AHI and CHI patients with a well-matched HIV negative MSM control group in China. Our study revealed that low levels of TC, HDL-C and LDL-C occurred in both AHI and CHI groups. High levels of AIP were prevalent during the early stages of HIV-1 infection. The level of TG, the TC/HDL-C ratio and proportion of dyslipidemia were, however, similar in all groups. The AIP correlated with TC, LDL-C, weight, BMI and DBP in MSM control group and correlated with weight, BMI and HIV RNA in the HIV infection group. The significant predictors of AIP were TG, HDL-C and acute HIV infection status.

Our results were similar with other studies which documented significantly lower levels of TC, HDL-C and LDL-C cholesterol in treatment-naïve HIV-infected individuals relative to seronegative controls [21, 22]. Among all the lipid parameters in treatment-naïve HIV-infected patients, TG appeared to have an inconsistent report. In our study, we found that the levels of TG between the HIV infection group and the control group had no statistically significant differences. This result was similar as a previous study [21]. However, Nguemaim NF and Daniyam CA demonstrated that HIV-infected patients had significantly lower TC, HDL-C but higher levels of TG [22, 23]. A possible explanation for the TG difference may be the differed degrees of immunosuppression of the HIV infection patients. A study conducted in Nigeria showed that the TG levels were reversely correlated with CD4 counts and positively correlated with viral load [11]. The HIV infected participants in our study had a better immune status as reflected by a median CD4 cell count of 404.0 (281.5–531.5) cell/μl in the AHI group and 173.0 (104.3–243.2) in the CHI group compared to previous studies. Increased TG tends to occur with profound immunosuppression. HIV infection induced a typically early decrease of TC first, followed by HDL-C and LDL-C, and TG increase last. These lipids alterations associated with specific changes in immune function [11, 21].

This was the first study that we knew of to report data on AIP in the acute HIV-infected individuals. The use of the AIP as an index of dyslipidaemia showed that the AHI patients were at higher risk of atherosclerosis compared with the MSM control group despite their low levels of TC and LDL-C. In our study, the AIP values between CHI group and MSM control group were not statistically different. Cajetan C. Onyedum’s study showed that higher AIP was frequent in ART-naïve CHI patients [24]. However, this study did not have a control group, and most of enrolled patients were female. Significantly more females than males had dyslipidemia, which may contribute to higher AIP value [11, 25]. Our result suggested that the patients in the early HIV infection stage should be monitored lipids frequently. China is the largest developing country and the economy develops imbalanced. Medical resource backwardness exists in remote areas. In resource limited setting of China, the AIP is a simple and practical ratio to estimate the risk of atherosclerosis and cardiovascular disease.

In terms of the relationship between AIP and other factors, our study showed that the AIP was closely related with factors such as TC, LDL-C, weight, BMI and DBP in the MSM group. We also demonstrated a relationship between AIP and HIV viral load level in the HIV infection group apart from weight and BMI. Those who had higher level of HIV RNA had a higher atherogenic risk profile. This is consistent with the previously reported studies which showed HDL-C was negatively correlated with viral load and the TG levels was positively correlated with viral load on the contrary [11, 22, 26]. These studies also demonstrated AIP or lipids parameters correlated with immunologic status of HIV infected people. In our study we did not find the association between AIP and CD4 cell counts of HIV infected people, further researches about this should be performed.

The result of multivariable linear regression demonstrated that acute HIV infection was associated with higher AIP after controlling for potential confounders. AHI patients had higher level of HIV viral load which may result in the increase of AIP. This may have reflected a direct relationship between viral replication and chronic inflammation caused by cytokine. The continuous viral replication induces inflammation and cytokine secretion such as IFN‑α, which was believed to make a contribution to increased TG level [27]. Janet Lo’s study showed that the high magnitude of viremia characteristic of AHI impaired cholesterol efflux from macrophages, which could be improved with suppression of viral replication by ART [13]. We speculate that suppressing viremia may reduce atherosclerotic risk in early stage of HIV infection and prospective analyses with larger sample size should be performed to confirm this. An urgent need exists for earlier dyslipidemia diagnosis and better access to treatment in China.

Strengths of our study include the well matched HIV negative MSM control group. Most studies used individuals from the general population as the healthy controls, who might have lifestyles and related risk factors differ from those of the HIV infected patients. Therefore, to precisely evaluate the effect of HIV infection on lipids metabolism and AIP among MSM, it is necessary to use HIV-negative MSM population as the control group. Moreover, due to the rising cost and the relative low HIV testing rate, screening for acute HIV infection is difficult in China. In this study, acute HIV infection patients were enrolled based on rigorous criteria.

There were several limitations in our study. First of all, the sample size of HIV infection group was too small to show a real difference and detect significant associations between AIP and other risk factors. Secondly, all the participants were MSM, our conclusion cannot be applied to other populations. Thirdly, due to the cross-sectional design, we could not infer causal relationships and assess the kinetics of AIP changes.

Despite these limitations, to our knowledge, this is the first study that investigated AIP among ART-naïve MSM patients in China and will no doubt stimulate more research in this field. As a convenient and cheap method to predict cardiovascular disease risk, AIP is recommended in China. Life style modification and prompt treatment are needed to prevent lipids metabolism dysfunction. Longitudinal studies are needed to evaluate the risk of atherosclerosis and cardiovascular events over time.

## Conclusions

Our study revealed that low levels of TC, HDL-C, LDL-C and high level of AIP were prevalent during the early stages of HIV-1 infection. The AIP correlated with TC, LDL-C, weight, BMI and DBP in MSM control group and correlated with weight, BMI and HIV RNA in the HIV infection group. The significant predictors of AIP were TG, HDL-C and acute HIV infection status. Life style modification and prompt treatment are needed to prevent lipids metabolism dysfunction.

## Abbreviations

AHI: Acute human immunodeficiency virus infection
AIDS: Acquired immune deficiency syndrome
AIP: Atherogenic index of plasma
ART: Antiretroviral therapy
BMI: Body mass index
CHI: Chronic human immunodeficiency virus infection
DBP: Diastolic blood pressure
FBG: Fasting blood glucose
HDL-C: High-density lipoprotein cholesterol
HIV: Human immunodeficiency virus
IQR: Interquartile range
LDL-C: Low-density lipoprotein cholesterol
MSM: Men who have sex with men
SBP: Systolic blood pressure
SD: Standard deviation
TC: Total cholesterol
TG: Triglycerides
VCT: Voluntary counseling and testing
WHO: World Health Organization
